# Intranasal Oxytocin Attenuates Cognitive Impairment, β-Amyloid Burden and Tau Deposition in Female Rats with Alzheimer’s Disease: Interplay of ERK1/2/GSK3β/Caspase-3

**DOI:** 10.1007/s11064-022-03624-x

**Published:** 2022-05-20

**Authors:** Samar O. El-Ganainy, Omar A. Soliman, Aya A. Ghazy, Maram Allam, Aya I. Elbahnasi, Amira M. Mansour, Mennatallah A. Gowayed

**Affiliations:** 1 Department of Pharmacology and Therapeutics, Faculty of Pharmacy, Pharos University in Alexandria, Alexandria, Egypt; 2 Department of Clinical Pharmacy, Faculty of Pharmacy, Kafrelsheikh University, Kafrelsheikh, Egypt; 3 Department of Pathology, Faculty of Medicine, Alexandria University, Alexandria, Egypt

**Keywords:** Oxytocin, Alzheimer, β-Amyloid, Cognitive impairment, Tau

## Abstract

Oxytocin is a neuropeptide hormone that plays an important role in social bonding and behavior. Recent studies indicate that oxytocin could be involved in the regulation of neurological disorders. However, its role in modulating cognition in Alzheimer’s disease (AD) has never been explored. Hence, the present study aims to investigate the potential of chronic intranasal oxytocin in halting memory impairment & AD pathology in aluminum chloride-induced AD in female rats. Morris water maze was used to assess cognitive dysfunction in two-time points throughout the treatment period. In addition, neuroprotective effects of oxytocin were examined by assessing hippocampal acetylcholinesterase activity, β-amyloid 1–42 protein, and Tau levels. In addition, ERK1/2, GSK3β, and caspase-3 levels were assessed as chief neurobiochemical mediators in AD. Hippocampi histopathological changes were also evaluated. These findings were compared to the standard drug galantamine alone and combined with oxytocin. Results showed that oxytocin restored cognitive functions and improved animals’ behavior in the Morris test. This was accompanied by a significant decline in acetylcholinesterase activity, 1–42 β-amyloid and Tau proteins levels. Hippocampal ERK1/2 and GSK3β were also reduced, exceeding galantamine effects, thus attenuating AD pathological hallmarks formation. Determination of caspase-3 revealed low cytoplasmic positivity, indicating the ceasing of neuronal death. Histopathological examination confirmed these findings, showing restored hippocampal cells structure. Combined galantamine and oxytocin treatment showed even better biochemical and histopathological profiles. It can be thus concluded that oxytocin possesses promising neuroprotective potential in AD mediated via restoring cognition and suppressing β-amyloid, Tau accumulation, and neuronal death.

## Introduction

Alzheimer’s disease (AD) is the most common neurodegenerative disease and the leading cause of all types of dementia [1]. AD cases increased steeply with more than doubling incidence in the last 20 years and are expected to reach more than 132 million people by 2050 [2]. Pathologically, the chief pathological hallmarks of AD are extracellular β-amyloid protein and intracellular neurofibrillary tangles precipitation [1]. The increase in disease burden requires innovative therapeutic options as all available medications slow the disease development rather than provide a cure [3].

Oxytocin (OX) is a neuropeptide that has a fundamental role in inducing uterine contractions and lactation [4]. In the brain, OX can act as a neuromodulator and a neurotransmitter [5]. Its role in the CNS became an attractive field of research, where preclinical and clinical studies indicate that OX neurotransmission could be involved in the regulation of various neurological disorders including schizophrenia [6], depression [7], autism [8], anxiety [9], and some forms of dementia [10]. The nasal application had been previously reported as a successful route to bypass the blood–brain barrier and deliver therapeutics in adequate amounts to the brain [11]. More specifically, intranasal OX administration was found to deliver relevant concentrations of OX to the brain, through channels surrounding trigeminal and olfactory nerve fibers. Following intranasal application, oxytocin accumulates in brain regions highly expressing OX receptors, including the hippocampus, in both humans and rats [12, 13].

Previous studies had shown promising outcomes of oxytocin treatment in neuroinflammatory and neurodegenerative models. Erbas et al. [14] reported that OX rescued neurons from apoptotic death fate in a rotenone rat model. Moreover, OX exerted an anti-inflammatory effect and attenuated microglial activity in the lipopolysaccharide-induced inflammatory model [15]. In an Experimental Stroke Model, treatment with OX had shown a neuroprotective potential via suppression of apoptotic signaling hallmarks [16]. The potential effect of OX on cognition functions was tested in rats subjected to stress and showed enhanced hippocampal plasticity following single or triple intranasal oxytocin dose [17, 18]. However, the possible effect of OX on AD-induced memory impairment and pathology has never been elucidated.

Aluminum is a profuse metal, highly existing in human surroundings, getting into the human body through food, water, drugs, and utensils. The ability of aluminum to accumulate in sensitive areas of the brain like the hippocampus and frontal cortex made it a possible main contributing factor in the pathogenesis of neurodegenerative disorders [19, 20]. Aluminium-mediated neurodegeneration has been well established as an experimental model of AD [21, 22], associated with elevated β-amyloid deposition [23], diminished cholinergic transmission, overexpression of phosphorylated Tau [24], and neuronal death [25]. Such a cascade of events in the brain results in cognitive dysfunction resembling AD patients [26].

Taking into account the aforementioned findings, this study investigated, for the first time, the possible effect of chronic intranasal oxytocin treatment on cognitive impairment in an aluminum chloride-induced AD in female rats. Restoration of memory functions was tested using Morris water maze at two-time intervals of treatment. The neuroprotective potential of OX was examined by assessing acetylcholinesterase enzyme activity, as well as β-amyloid and Tau deposition. In addition, ERK1/2, GSK3β, and caspase-3 levels were assessed as neurobiochemical mediators in AD. A histopathological examination of rats’ hippocampi was performed. Those effects were compared to the standard drug, galantamine. In addition, the possible synergistic potential when combining both oxytocin and galantamine was also addressed.

## Experimental Procedures

### Chemicals

Both oxytocin and galantamine were purchased from Sigma Aldrich (St. Louis, USA). Aluminum chloride (AlCl_3_) was purchased from El-Gomhouria chemicals (Alexandria, Egypt).

### Animals and Ethical Considerations

Female Sprague Dawley rats (180–210 g); 8–9 weeks of age; were obtained from the Animal House Unit facility at the Faculty of Pharmacy, Pharos University in Alexandria (Alexandria, Egypt). Animals were kept under controlled conditions of room temperature (25 ± 2 °C), humidity (60 ± 10%) and maintained on a 12-h light/dark cycle. Animals were kept 5 per cage with free access to food and water, using a standard laboratory diet. All experimental animals were approved by the Institutional “Research Ethics Approval Committee” of the Faculty of Pharmacy, Pharos University in Alexandria, Egypt (PUA 01202101033019) and comply with ARRIVE guidelines and the National Research Council’s Guide for the Care and Use of Laboratory Animals.

### Experimental Design

**Fig. 1:**
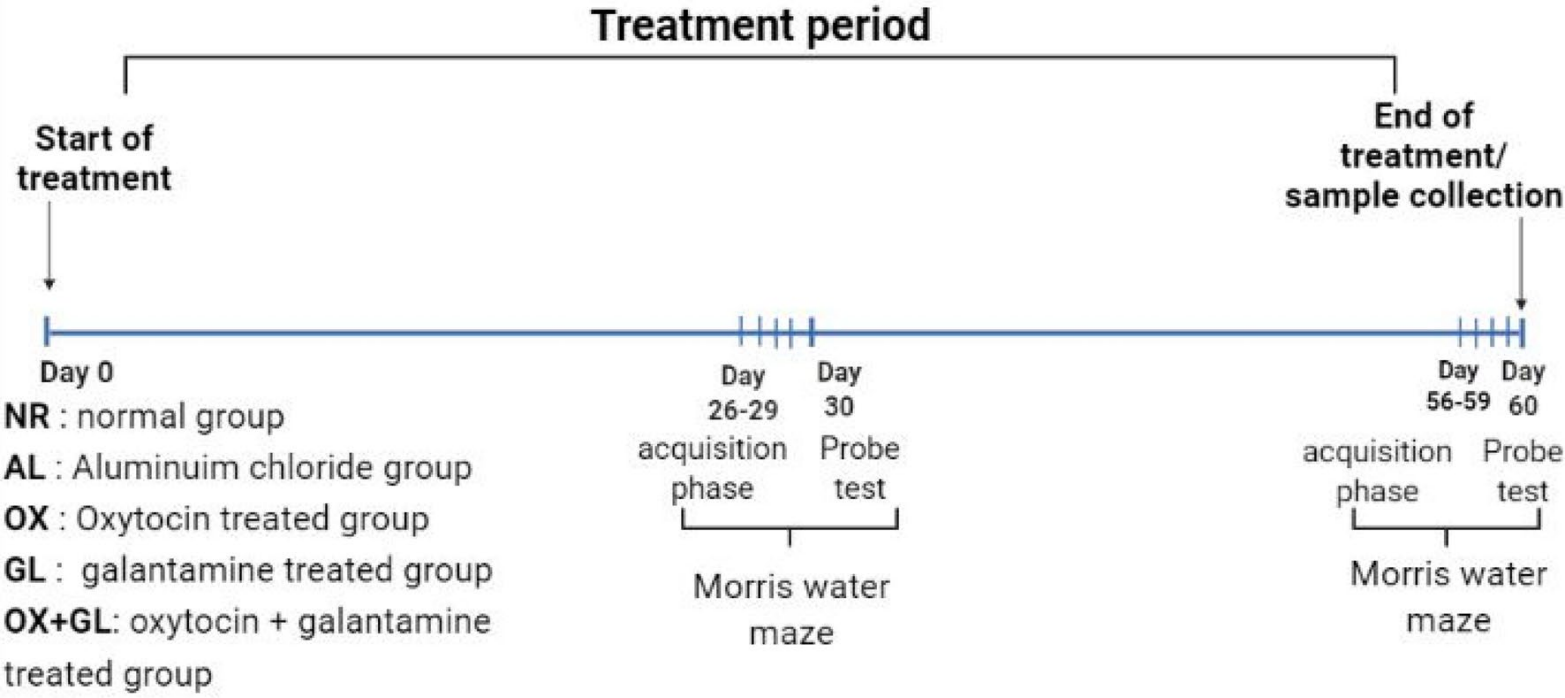
Experimental timeline showing different treatments and Morris water maze time points

Rats were randomly divided into five groups (n = 8) and treated daily for 8 consecutive weeks as follows: (1) *NR*: normal control group, receiving a daily oral dose of water, (2) *AL*: AD-model group, in which rats were receiving a daily oral dose of aluminum chloride (100 mg/kg), (3) *OX*: OX-treated group, in which rats received an oral dose of aluminum chloride (100 mg/kg) and intranasal dose of oxytocin (1.25 IU/kg) [27] (4) *GL*: GL-treated group, received oral doses of aluminum chloride (100 mg/kg) and galantamine (3 mg/kg), (5) *OX* + *GL*: OX-GL-treated group, received an oral dose of aluminum chloride (100 mg/kg), oral galantamine (3 mg/kg) and intranasal dose of oxytocin (0.25 IU/kg) (Fig. 1).

Female rats were used to establish the Aluminium mediated Alzheimer’s model, as the female sex are more vulnerable to developing AD, deposition of plague, and progression of disease pathology [28]. Moreover, OX affects multiple pathways in the female brain [29], nominating them as candidate sex for screening of anti-Alzheimer potential. The aluminum chloride dose was selected according to previous reports despite being higher than the human dose. However, this dose mimics molecular, behavioral, biochemical, and neuronal discrepancies similar to AD in humans [23, 25].

All treatments were administered 60 min apart from aluminum chloride dosing [30, 31]. Intranasal OX dose was selected with reference to previous works, showing that this dose accumulates sufficiently in rat brain regions including the hippocampus, with minimal peripheral concentration [13, 27], without toxicological manifestations [32]. Under light ether anesthesia, oxytocin was administered via the intranasal route (20 µl per 200 g rat), in one nostril using a micropipette. After intranasal application, rats were held in an upright position for 10 s to allow the flow of solution with normal inhalation.

Morris water maze test was conducted on days 26–30, as well as at the end of the experiment on days 56–60, as described in Fig. 1. At the end of the experiment, rats were euthanatized using an overdose of phenobarbital, brains were harvested and divided into halves. The first half was fixed in formol/saline (10%) while the hippocampus was dissected from the other brain half and stored at − 80 °C for further biochemical analysis.

### Morris Water Maze

Morris water maze was used to test spatial learning and memory, at two-time points; day 30 (mid-treatment) and day 60 (end-treatment). A dark circular tank divided into four equal quadrants was used and a platform was placed in the target quadrant submerged 1 cm below the water surface. During the acquisition phase, rats were trained for 4 consecutive days, 30 min after drug dosing. Rats were allowed to swim until locating the platform and allowed to stay on it for 10 s. When a rat failed to locate the platform during 120-s trials, it was gently guided to it and left there for 10-s. Each rat was subjected to three daily training sessions with 5-min inter-trial intervals. The same conditions were applied during the first (days 26–29) and the second (days 56–59) acquisition phases.

Escape latency, defined as the average total time taken in each daily trial of the acquisition phase, was calculated. During the probe test, the platform was removed and rats were released from the opposite position for 60 s. The index of retrieval, which is the time the rat spent swimming within the target quadrant searching for the platform, was recorded. In addition, the time spent to enter the target quadrant, i.e. entrance latency, and the frequency of passing through the target quadrant during the retention phase were recorded [33].

### Neurobiochemical Parameters

Quantitative determination of the following parameters was performed in hippocampi homogenates using rat-specific ELISA kits according to the manufacturer’s instructions; acetylcholinesterase (AChE) activity (Sigma-Aldrich, USA), fibrillar β-amyloid 1–42 level, and tau (Novusbio, USA) as well as ERK1/2 and GSK3β levels (MyBiosource, USA). Acetylcholinesterase (AChE) activity was expressed as µmole/min/mg protein, β-amyloid 1–42 level, and Tau were expressed as pg/mg protein, while ERK1/2 and GSK3β were expressed as ng/mg protein. Protein content in each sample was quantified using the biuret method [34]. Both the right and left hippocampus were used for the protein determination.

### Histopathological & Immunohistochemistry Examination

From all experimental groups, the left side of the brain was excised and washed with cold phosphate-buffered saline followed by fixation using 10% formalin. Paraffin-embedded blocks were prepared and sectioned. Sections were stained with Haematoxylin and Eosin (H&E) and examined using a light microscope. Under the light microscope, the brain was examined for any histopathological changes with emphasis on the hippocampus (CA1 & CA3) and cerebral cortex regions.

For immunohistochemical examination, sections at the level of the hippocampus on coated slides were obtained from the prepared paraffin blocks. Sections were first deparaffinized using xylene, and descending concentrations of ethanol (100%, 95%, and 70%) for 3 min at each step. After deparaffinization, antigen retrieval was carried out. Then slides were washed with 1% BSA with gentle agitation. The sections were then blocked in 10% normal serum with 1% BSA in TBS and incubated for 2 h for room temperature. Sections were then incubated in primary antibody; anti-rat Caspase 3 (a marker of apoptosis), clone 9H19L2, monoclonal antibody (1:50) in TBS with 1% BSA overnight at 4 °C. After washing with TBS containing 0.025% triton 100× with gentle agitation the sections were incubated with anti-mouse HRP IgG conjugated antibody (1:40,000) in TBS with 1% BSA for 2 h at room temperature. Immunoreactivity was visualized after incubation with DAB for 10 min at room temperature followed by hematoxylin staining for 10 min and dehydration in the alcohol series using an Olympus microscope at high magnification. The percent of positively stained cells were counted using computer-assisted image analysis software (Leica Application Suite v4.12.0; Leica Microsystems, Switzerland). Immunohistochemically stained sections were scored according to the intensity of staining (0 = negative, 1 = mild, 2 = moderate, 3 = intense positivity) [35].

### Statistical Analysis

Data obtained were presented as mean ± S.E.M (n = 8). Results were analyzed using one-way ANOVA followed by Student–Newman–Keuls multiple comparison test. Entrance latency was subjected to two-way ANOVA followed by the Bonferroni test, where the two factors considered were treatment and time. Statistical analysis was performed using GraphPad Prism software (version 5.0). For all the statistical tests, the level of significance was fixed at p < 0.05.

## Results

### Oxytocin Effect on Memory Retention in Morris Water Maze

**Fig. 2:**
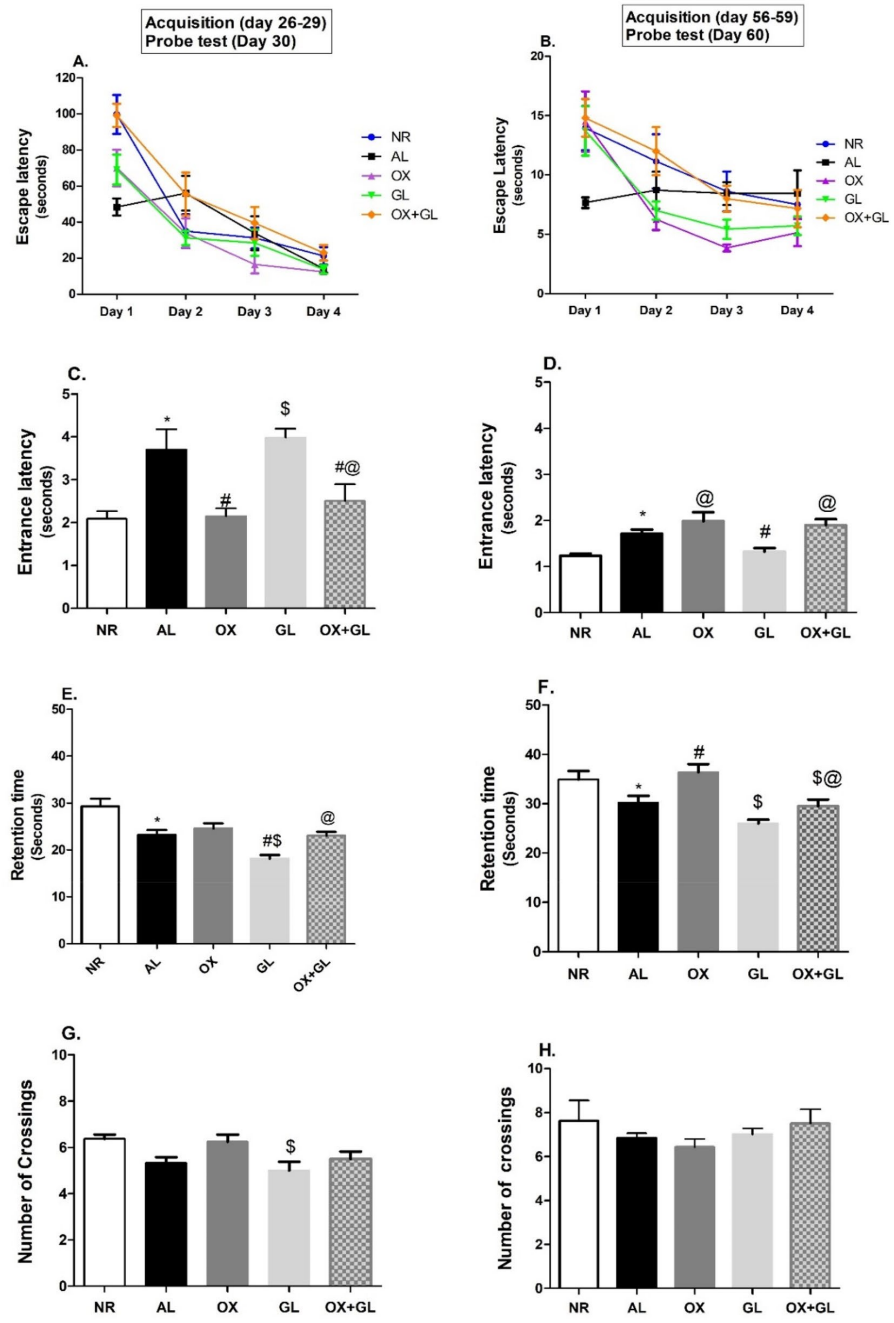
Effect of oxytocin, galantamine, and combined treatment on Morris water maze parameters in aluminium-induced AD model: **A**, **B** Escape latency, **C**, **D** Entrance latency (Day 30), **E**, **F** Retention time, **G**, **H** Number of crossings. Statistical analysis was done using one-way ANOVA followed by Student–Newman–Keuls multiple comparison test. Two-way ANOVA was used to test the effect of treatment and time in escape latency followed by Bonferroni test. Data are presented as mean ± S.E.M (n = 8); *p < 0.05 vs. control, ^#^*p* < 0.05 vs AlCl_3_ group, ^$^*p* < 0.05 vs. OX group and ^@^*p* < 0.05 vs. GL group. *NR* normal; *AL* aluminum chloride; *OX* oxytocin; *GL* galantamine

In the Morris water maze test, escape latency of the AL group showed a slow declining rate starting at day 3 compared to normal rats at first acquisition, reflecting defects in spatial learning capabilities. At the second acquisition, AL-treated rats exhibited a plateauing performance denoting progressive loss of spatial learning over time, (Fig. 2A, B). On the other hand, rats treated with OX, GL, and OX + GL showed a rapid decline in escape latency at the two acquisitions, noting that OX & GL groups surpassed their combined treatments (Fig. 2A, B). Two-way ANOVA analysis revealed a significant effect of time (*F* = 48.97 *p* < 0.0001, *F* = 23.48 *p* < 0.0001), treatment (*F* = 5.95 *p* = 0.0003, *F* = 5.96 *p* = 0.001) and interaction (*F* = 8.28 *p* = 0.0034, *F* = 10.26 *p* = 0.029) in the two acquisition trials, respectively.

Concerning the probe test, the AL group exhibited a significantly longer time entering the target quadrant compared to NR, i.e., higher entrance latency at day 30 (*F* (4,35) = 8.16, *p* < 0.0001) and day 60 test (*F* (4,35) = 8.3, *p* < 0.0001). OX treatment showed the lowest significant latency to enter the target quadrant compared to other treatments at day 30, reflecting its superior effect on memory regain. However, at the day 60 probe trial, GL-treated rats surpassed OX-treated rats, as they entered the target quadrant more rapidly than other treatments. Results are presented in (Fig. 2C, D).

Regarding retention time, NR had the longest stay period in the right quadrant across all groups, while the AL group showed the shortest retention time compared to normal rats (Fig. 2E, F). On day 30, nearly equal retention time was noted among all treatments except for GL-treated rats which showed an even shorter time of retention when compared to the AL group, (*F* (4,35) = 13.8, *p* < 0.0001) (Fig. 2E). At day 60 probe trial, OX-treated rats exhibited the highest significant retention time compared to other treatments (*F* (4,35) = 9.19, *p* < 0.0001), nearly equal to NR rats (Fig. 2F).

Also, the AL group showed the lowest number of crossings over the non-existing platform compared to normal rats. A number of crossings of all other treatments were nearly equal to the AL group except for OX and OX + GL group showing a slight insignificant increase in crossing frequency at day 30, (Fig. 2G), while the OX + GL group has shown the highest number of crossings over the non-existing platform at day 60 (Fig. 2H).

### Oxytocin Decreased Acetylcholinesterase and β-Amyloid 1–42 Production in AlCl_3_ Induced AD Model

**Fig. 3:**
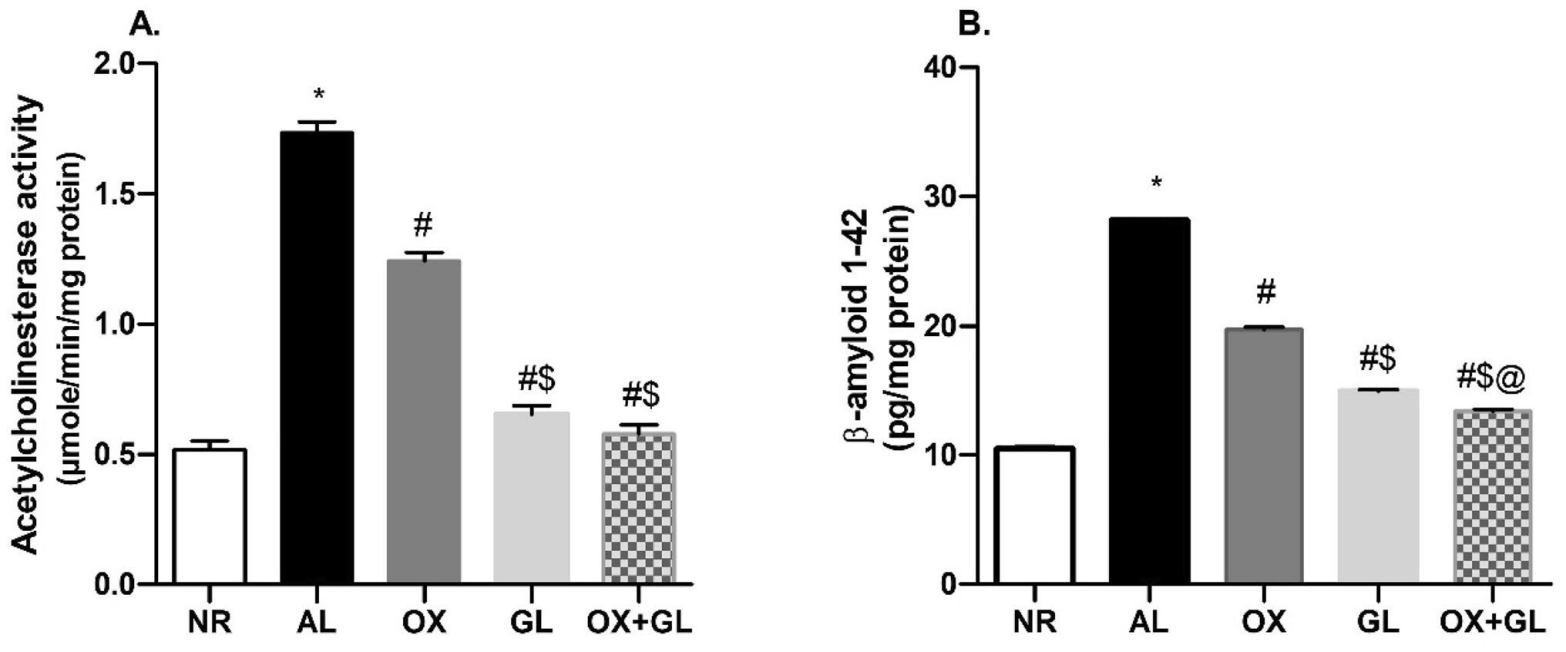
Effect of oxytocin, galantamine, and combined treatment on aluminum chloride-induced changes in **A** Acetylcholinesterase activity, **B** β-amyloid 1–42 levels. Quantitative determination was performed in hippocampi homogenates using rat-specific ELISA kits. Statistical analysis was done using one-way ANOVA followed by Student–Newman–Keuls multiple comparison test. Data are presented as mean ± S.E.M (n = 8); *p < 0.05 vs. control, ^#^*p* < 0.05 vs AlCl_3_ group, ^$^*p* < 0.05 vs. OX group and ^@^*p* < 0.05 vs. GL group. *NR* normal; *AL* aluminum chloride; *OX* oxytocin; *GL* galantamine

AL induced AD group showed markedly increased AChE activity as well as β-amyloid 1–42 level in rats’ hippocampi (*F* (4,35) = 1775, *p* < 0.0001). OX treatment induced a significant decline in both AChE activity, and β-amyloid 1–42 levels compared to the AL group (Fig. 3A, B). Moreover, GL treatment showed a substantial decline in AChE activity and β-amyloid 1–42 level compared to both AL and OX groups (*F* (4,35) = 1612, *p* < 0.0001). Similar results were obtained in OX + GL treated group with no significant difference concerning GL-treated rats.

### Oxytocin Reduced ERK1/2, GSK3B, and Tau Levels in AlCl_3_ Induced AD Model

**Fig. 4:**
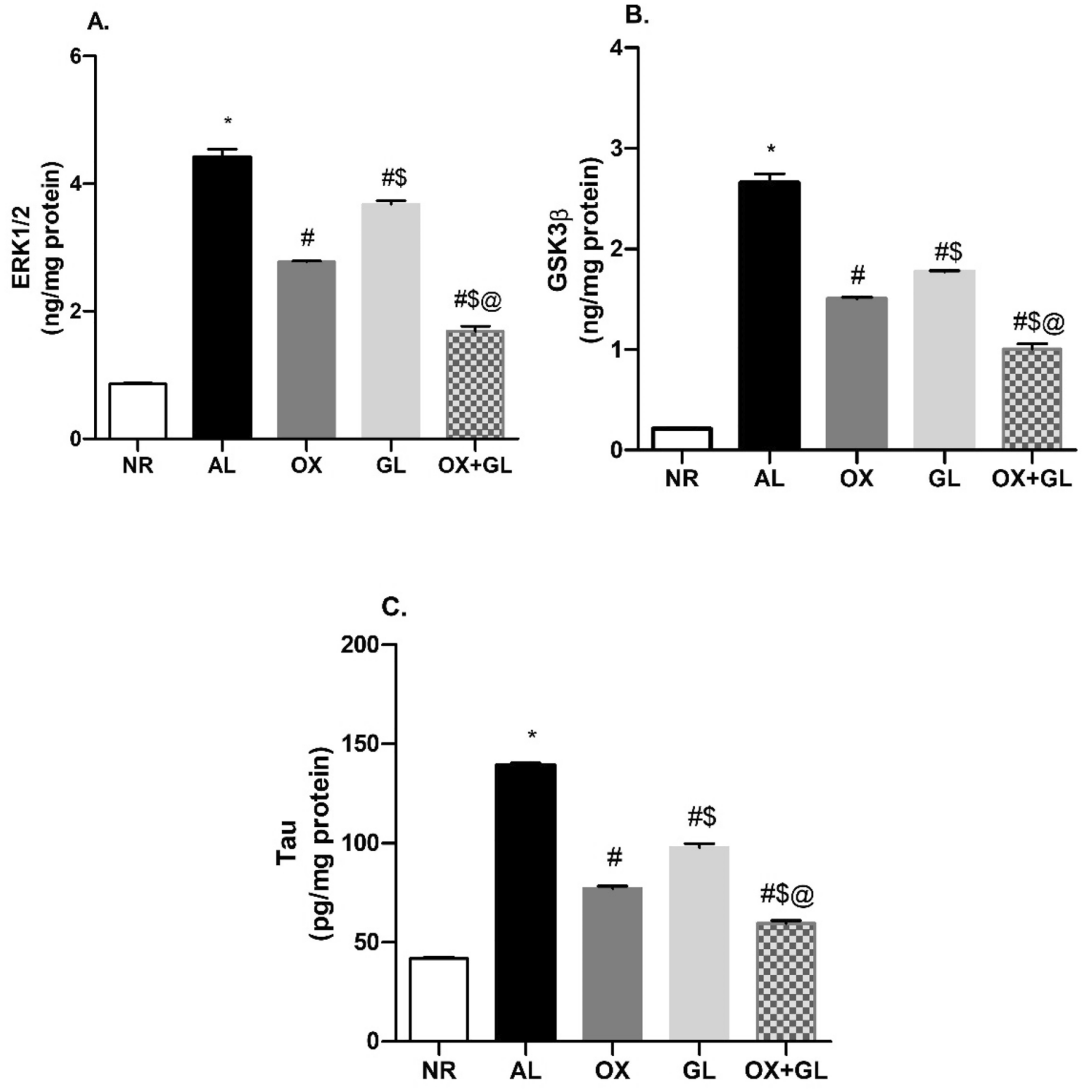
Effect of oxytocin, galantamine, and combined treatment on aluminum chloride-induced changes in; **A** ERK1/2 level, **B** GSK3β level, **C** Tau protein content. Quantitative determination was performed in hippocampi homogenates using rat-specific ELISA kits. Statistical analysis was done using one-way ANOVA followed by Student–Newman–Keuls multiple comparison test. Data are presented as mean ± S.E.M (n = 8); *p < 0.05 vs. control, ^#^*p* < 0.05 vs AlCl_3_ group, ^$^*p* < 0.05 vs. OX group and ^@^*p* < 0.05 vs. GL group. *NR* normal; *AL* aluminum chloride; *OX* oxytocin; *GL* galantamine

As shown in Fig. 4A ERK1/2 level was prominently increased in the AL group compared to normal rats. While OX as well as GL-treated rats exhibited a significant decline in hippocampal ERK1/2 level compared to the AL group. However, the OX + AL group showed the highest decline compared to all other groups (*F* (4,35) = 418, *p* < 0.0001).

Similarly, AL induced a considerable rise of GSK3β levels with respect to normal rats (*F* (4,35) = 383.6, *p* < 0.0001). While OX or GL treatment revealed a decrease in GSK3β levels compared to the AL group. The combined treatment of OX + GL showed the least GSK3β content compared to all other groups, as presented in (Fig. 4B).

Tau levels were significantly increased in the AL group compared to the NR group (*F* (4,35) = 770.4, *p* < 0.0001), as shown in Fig. 4C. Both OX and OX + GL treated groups showed reduced Tau content compared to the AL group and GL treated group. However, OX + GL showed a near-normal Tau content surpassing all other groups.

### Oxytocin Reverted Histopathological Insults and Suppressed Caspase-3 Activity

**Fig. 5:**
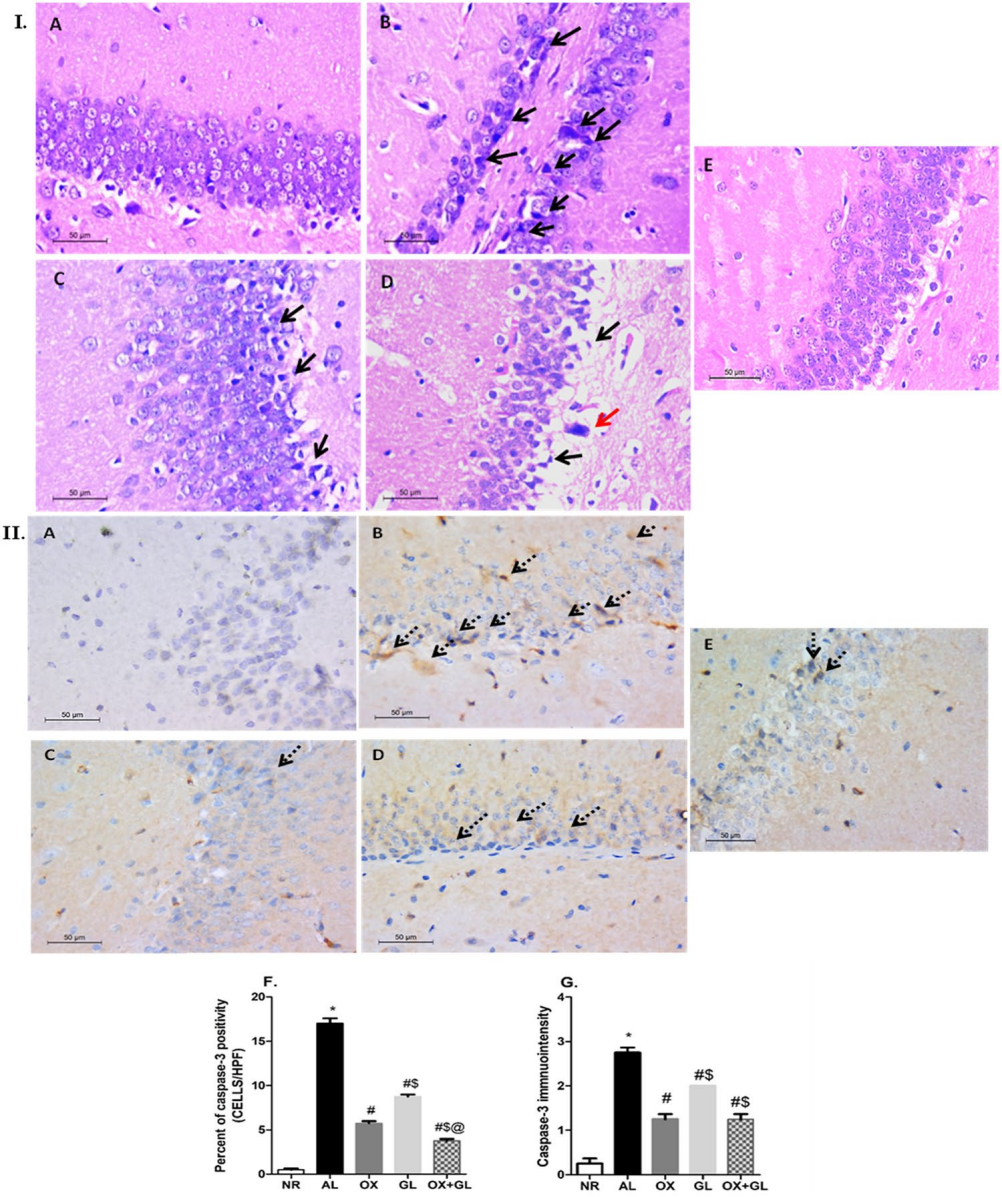
**I** Photomicrographs of rats’ hippocampi (H&E, ×400) and **II** Caspase-3 immunohistochemically stained section of rats’ hippocampi (anti caspase-3 antibody, ×400); (A) normal group, (B) ALCl_3_ group, (C) oxytocin-treated group, (D) galantamine-treated group, (E) combined galantamine and oxytocin-treated group. *Black arrow; neuronal degeneration, red arrow; pyknotic nuclei, dashed arrow; positive cytoplasmic staining*. Statistical analysis was done using one-way ANOVA followed by Student–Newman–Keuls multiple comparison test. Data are presented as mean ± S.E.M (n = 8); *p < 0.05 vs. control, ^#^*p* < 0.05 vs AlCl_3_ group, ^$^*p* < 0.05 vs. OX group and ^@^*p* < 0.05 vs. GL group. *NR* normal; *AL* aluminum chloride; *OX* oxytocin; *GL* galantamine

Morphological changes were evaluated in rats’ hippocampal tissue to detect any damage to neurons. In the control group, the hippocampal pyramidal layers showed average thickness in which cells were arranged in parallel layers, showing vesicular nuclei (Fig. 5I A). In AL-treated rats, hippocampal pyramidal cells showed severe neuronal degeneration with marked shrunken dark basophilic neurons in the polymorphic and pyramidal layers especially in Cornu Ammonis 1 (CA1). There was a remarkable decrease in the width of the pyramidal cell layer with the disorderly cellular arrangement, (Fig. 5I B). In groups receiving either GL or OX, the hippocampal pyramidal cells were orderly arranged, cell morphology was significantly improved presenting a reduced number of cells with pyknotic nuclei. Some cells showed cell edema, and the pyramidal cell layer thickness was nearly normal, as shown in (Fig. 5I C, D respectively). In OX + GL rats, the hippocampal damage was almost totally reversed and pyramidal cells were neatly arranged. Nearly all the pyramidal cell nuclei were vesicular with rare pyknotic nuclei, and the pyramidal cell layer was of an average thickness (Fig. 5I E).

Regarding caspase-3 immunohistochemically stained sections of the normal group, hippocampi revealed negative staining. On the other hand, sections of the AL group showed cytoplasmic positivity in a large number of the pyramidal cells (Fig. 5II B), indicating accelerated neuronal death. Generally, GL or OX treatments revealed cytoplasmic positivity in a few cells in the hippocampal pyramidal layer (Fig. 5II C, D). In the OX + GL group very low number of positively stained pyramidal cells was observed (Fig. 5II E). Quantitatively, caspase-3 immunopositivity was significantly higher in the AL group compared to the control. In the OX group, the level of caspase-3 was prominently halted compared to the AL group or GL group, (*F* (4,35) = 404.1, *p* < 0.0001). The OX + GL group showed an even lower level of caspase-3, as shown in Fig. 5I F. While the GL group showed moderate to low caspase-3 immunopositivity (*F* (4,35) = 81.6, *p* < 0.0001). The OX and OX + GL group showed nearly equal immunopositivity, exhibiting overall lower scores than AL and GL groups (Fig. 5I).

## Discussion

Morris water maze is a benchmark behavioral test in assessing AD-associated memory impairment and its possible amendment by potential neurocognitive treatments [36]. In the current work, the AL group showed reproducibility when it comes to deteriorated learning and spatial memory capabilities shown in a slow decline of escape latency and diminished retention time, respectively. Such finding was previously reported in former studies [23, 30]. OX-treated rats revealed dominance over other treatments in restoring spatial memory capabilities, yet showing progressive effect over time only in retention ability. It is worth noting that the role of OX in long-term memory establishment has been previously reported during motherhood, with articulate involvement of the MAP kinase pathway [29]. Also, OX has been involved in inducing neurogenesis, which is reported to be impaired in AD models [37]. Combined OX and GL treatment showed slight memory restoration in the water maze test, manifested only in increased crossings and short entrance latency to the target quadrant.

As low ACh levels in the brain have been associated with memory decline in AD [38], inhibition of AChE and enhancement of the cholinergic transmission is considered the most promising therapeutic approach [39]. Treatment of AD rats with GL in the current study successfully reduced AChE activity, an effect that has been previously documented [40, 41]. Interestingly, the administration of OX also succeeded to decrease the activity of AChE compared to the AL group. Kocyigit et al. [42] have shown the ability of OX to decrease AChE activity in rats ‘liver and kidney, an effect that was antagonized by atosiban, the specific OX receptor antagonist. To our knowledge, no earlier studies investigated the effect of OX on AChE in the brain.

The most abundant type of β-amyloid in AD pathology is the β-amyloid 1–42, which is characteristic of cognitive impairment and considered a reliable biomarker of the AD [43, 44]. For the first time, we show here a promising effect of OX in decreasing β-amyloid 1–42 in rats’ hippocampi, an effect that has been potentiated when combined with GL. Recently, a study by Takahashi et al. [45] has shown the ability of OX to reverse the β-amyloid 25–35 induced impairment of synaptic plasticity in hippocampal slices of the mouse brain. Earlier, the oxytocin pathway has been reported as one of the protective mechanisms against β-amyloid toxicity, where atosiban administration enhanced the β-amyloid toxicity in SH-SY5Y cell line neurons [46].

The extracellular signal-regulated kinase1/2 (ERK1/2) pathway is known to be implicated in many neurodegenerative diseases including AD [47]. Sustained amplification of ERK1/2 plays an important role in neuronal death via caspase and non-caspase-dependent pathways [48]. Moreover, important crosstalk exists between ERK1/2 and the major pathological hallmarks of AD: β-amyloid and Tau phosphorylation, contributing to memory deficits [47]. Activation of this kinase promotes the phosphorylation of amyloid precursor protein enhancing β-secretase proteolysis and β-amyloid peptide accumulation [49]. Simultaneously, increased amyloid production further activates the ERK1/2 pathway promoting the neuronal death [50]. In this work, AlCl_3_ treated rats showed an upsurge in ERK1/2 levels parallel to β-amyloid accumulation, a finding that was previously documented [51]. Treatment with OX induced a significant decline in hippocampi ERK1/2 levels. While it is reported to induce ERK activation via its receptor stimulation [51], oxytocin previously inhibited ERK1/2 in LPS-induced neuroinflammation model [15] and autistic mice amygdala [52]. This differential effect could be in part referred to as oxytocin receptor localization within scaffolds proteins. It was reported that oxytocin could potentiate ERK activation when its receptor is localized inside caveolin-1-enriched microdomains, but reduced ERK1/2 when localized outside those microdomains [53]. Suppression of ERK1/2 levels in the current work adds to oxytocin’s potential to reverse neuronal death and memory impairment.

Glycogen synthase kinase 3 beta (GSK3β) is an important kinase in the pathogenesis of AD, contributing to amyloid deposition and neurofibrillary tangles formation [54]. Its role was highlighted with the development of GSK3 transgenic mice showing prominent degeneration in the dentate gyrus. Double transgenic mice (GSK3, Tau) also presented with severe memory impairment and neuronal degeneration [55]. In the current work, AlCl_3_ treated rats showed a substantial rise in hippocampi GSK3β levels, as was previously reported [23, 25]. Such increase is a causal consequence of amyloid deposition. GSK3β enhances the β-secretase enzyme, responsible for amyloid precursor protein cleavage during the amyloidogenic process [56]. In turn, increased amyloid deposition activates GSK3β pathway [57], inducing a vicious cycle of insulting mediators. Treatment with OX significantly attenuated hippocampal GSK3β rise. Such inhibition has been formerly implicated as a successful strategy to treat memory impairment and neurodegeneration in AD [58]. To our knowledge, this is the first study to report the suppressive effect of oxytocin on the brain’s GSK3β level. This finding could be referred to as the reported stimulatory effect of oxytocin on the AKT/PI3K pathway observed in many contexts [59, 60]. In AD brains, activated AKT is known to suppress GSK3β and its subsequent phosphorylation of Tau, thus providing a protective effect against neurodegeneration [61].

Given the upsurge of ERK1/2 and GSK3 levels in the adopted model, it can be deduced that the Tau protein level might subsequently increase. Hippocampi Tau levels exhibited a substantial rise following AlCl_3_ administration. Increased microtubule-associated Tau levels had been previously reported in the CSF, hippocampi, and cerebral cortices of AD models, correlating with neuronal death and cognitive impairment [62, 63]. Clinically, increased total Tau levels in patients CSF reflects disease intensity and progression [64]. Among various mediators, ERK1/2 & GSK3β are reported to promote Tau phosphorylation and to enhance neurofibrillary tangles formation [65]. Regarding its suppressive effect on both ERK1/2 and GSK3β levels, oxytocin treated group disclosed a substantial reduction in hippocampal Tau content. Earlier, it was shown that oxytocin upsurge induces an interplay of neuronal plasticity affecting microtubule-associated proteins including Tau [66].

Apoptosis is a major pathway activated during the progression of AD, leading to neuronal death [67]. Activated caspase-3, a chief executioner of caspase in the apoptotic process, has been reported in post mortem AD patient’s brain [68] as well as in AD animal models [69]. In the current model, AlCl_3_-induced an upsurge in caspase-3 activation in rats’ hippocampi, similar to findings of previous studies. Park et al. [70] had recently shown that localized caspase activation enhanced amyloid precursor protein cleavage leading to the accumulation of cytotoxic peptides and synaptic damage. In addition, caspase-3 is correlated with cleavage and truncation of Tau, facilitating tangles formation [71]. Oxytocin treatment suppressed caspase-3 activation, an effect that was similarly reported in other neurodegenerative disorders [14]. These findings were parallel to the histopathological findings revealing restored pyramidal layer arrangement and thickness in OX, GL as well as combined treated group.

Interestingly, combined oxytocin and galantamine treatment showed better biochemical & histopathological profiles when compared to each drug alone. A synergetic effect was observed on the decline in ERK1/2, GSK3β, and caspase-3 levels as well as both amyloid protein and Tau content. This could be attributed to the superior effect of each drug alone on a specific set of markers, thus culminating in the combined treatment group into synergistic outcomes. However, this was not clearly translated into the behavioral testing. An effect that could be attributed to additional unveiled mechanisms of OX in enhancing cognition in the AD model.

## Conclusion

**Fig. 6:**
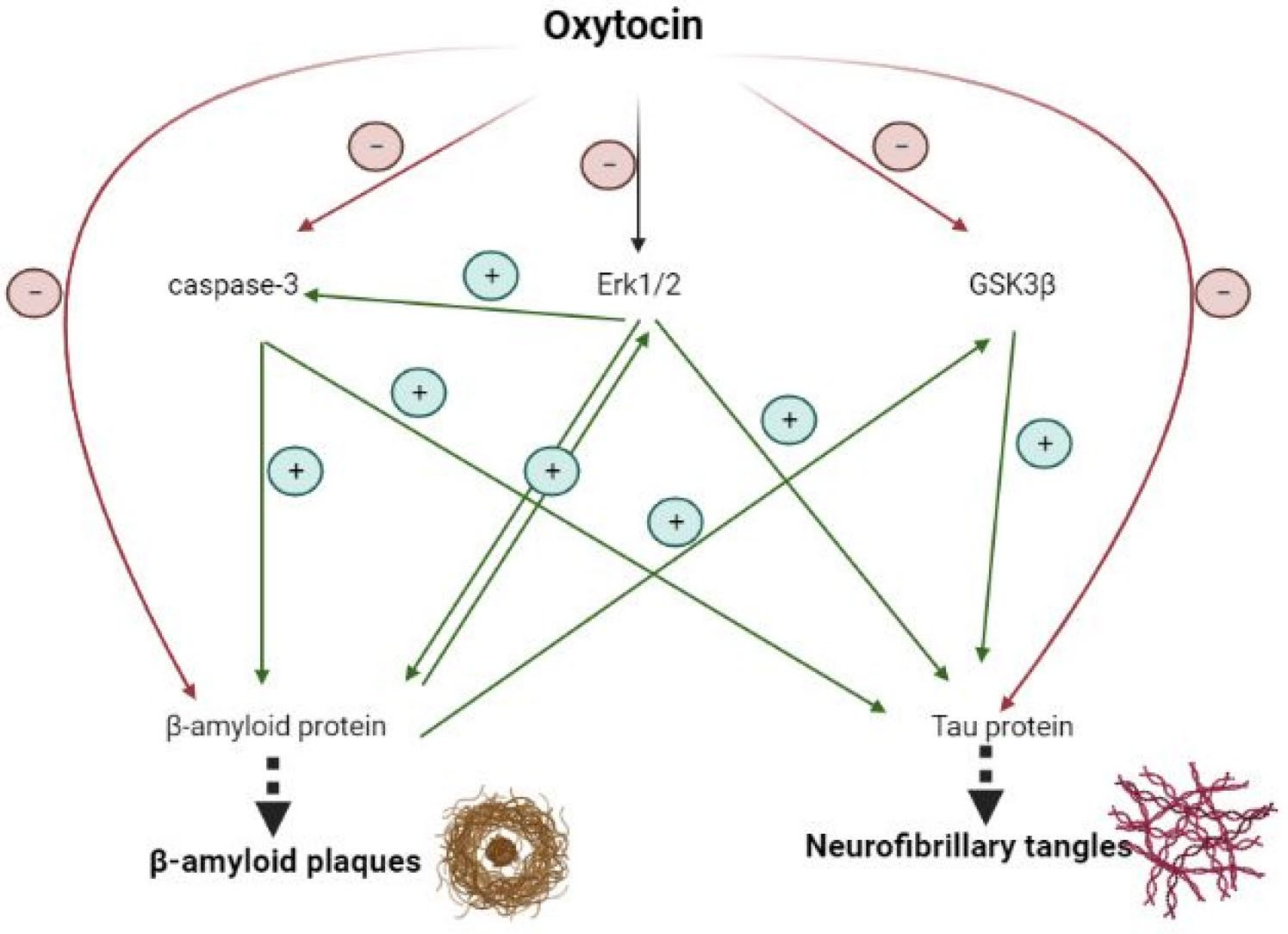
Possible protective mechanisms of oxytocin in AD hippocampus

This experimental work elucidated for the first time, the potential therapeutic effect of intranasal oxytocin on AD-induced in rats. This was manifested as improved cognitive behavior in the Morris water maze, accompanied by suppression of acetylcholinesterase activity, hippocampal β-amyloid deposition, and Tau levels. Oxytocin as well halted ERK1/2 and GSK3β kinases involved in the activation of pathological hallmarks of AD (Fig. 6). This was associated with diminished neuronal death and a restored histopathological profile. All previous findings support the novelty of the current work and encourage future studies to disclose the full mechanisms of oxytocin in reversing memory dysfunction and AD pathology.

## Data Availability

Enquiries about data availability should be directed to the authors.
